# Key mutations in the C-terminus of the HBV surface glycoprotein correlate with lower HBsAg levels *in vivo*, hinder HBsAg secretion *in vitro* and reduce HBsAg structural stability in the setting of HBeAg-negative chronic HBV genotype-D infection

**DOI:** 10.1080/22221751.2020.1757998

**Published:** 2020-05-13

**Authors:** Romina Salpini, Arianna Battisti, Lorenzo Piermatteo, Luca Carioti, Olympia E. Anastasiou, Upkar S. Gill, Domenico Di Carlo, Luna Colagrossi, Leonardo Duca, Ada Bertoli, Katia Yu La Rosa, Lavinia Fabeni, Alessandra Iuvara, Vincenzo Malagnino, Carlotta Cerva, Miriam Lichtner, Claudio M. Mastroianni, Giuseppe Maria De Sanctis, Maurizio Paoloni, Massimo Marignani, Caterina Pasquazzi, Nerio Iapadre, Giustino Parruti, Jacopo Vecchiet, Loredana Sarmati, Massimo Andreoni, Mario Angelico, Sandro Grelli, Patrick T. Kennedy, Jens Verheyen, Stefano Aquaro, Francesca Ceccherini-Silberstein, Carlo Federico Perno, Valentina Svicher

**Affiliations:** a Department of Experimental Medicine, University of Rome “Tor Vergata”, Rome, Italy; b Barts Liver Centre, Blizard Institute, Barts and The London SMD, QMUL, London, UK; c Institute of Virology, University-Hospital, University Duisburg-Essen, Essen, Germany; d Paediatric Clinical Research Center “Romeo and Enrica Invernizzi”, University of Milan, Milan, Italy; e Microbiology and Virology Unit, University of Milan, Milan, Italy; f Laboratory of Virology, National Institute for Infectious Diseases “Lazzaro Spallanzani” -IRCCS, Rome, Italy; g Microbiology and Virology Unit, Tor Vergata University Hospital, Rome, Italy; h Infectious Diseases Unit, Tor Vergata University Hospital, Rome, Italy; i Public Health and Infectious Disease Department, “Sapienza” University, Rome, Italy; j “Umberto I” University Hospital, Rome, Italy; k Infectious Disease Unit, “S.S. Filippo e Nicola” Hospital, Avezzano, Italy; l Department of Gastroenterology, “S. Andrea Hospital”, Rome, Italy; m “San Salvatore Hospital”, L’ Aquila, Italy; n Infectious Disease Unit, Pescara General Hospital, Pescara, Italy; o Department of Medicine and Science of Aging, Clinic of Infectious Diseases, University “G. d'Annunzio” Chieti-Pescara, Chieti, Italy; p Hepatology Unit, Tor Vergata University Hospital, Rome, Italy; q Department of Pharmacy, Health and Nutritional Sciences, University of Calabria, Rende, Italy; r Department of Oncology and Haemato-oncology, University of Milan, Milan, Italy

**Keywords:** HBeAg-negative infection, HBsAg levels, HBsAg C-terminus, HBsAg mutations, HBV genotypes

## Abstract

Increasing evidences suggest that HBsAg-production varies across HBV-genotypes. HBsAg C-terminus plays a crucial role for HBsAg-secretion. Here, we evaluate HBsAg-levels in different HBV-genotypes in HBeAg-negative chronic infection, the correlation of specific mutations in HBsAg C-terminus with HBsAg-levels *in-vivo,* their impact on HBsAg-secretion *in-vitro* and on structural stability *in-silico*.

HBsAg-levels were investigated in 323 drug-naïve HBeAg-negative patients chronically infected with HBV genotype-D(*N* = 228), -A(*N* = 65) and -E(*N* = 30). Genotype-D was characterized by HBsAg-levels lower than genotype-A and -E (3.3[2.7–3.8]IU/ml; 3.8[3.5–4.2]IU/ml and 3.9[3.7–4.2]IU/ml, *P* < 0.001). Results confirmed by multivariable analysis correcting for patients’demographics, HBV-DNA, ALT and infection-status.

In genotype-D, specific C-terminus mutations (V190A-S204N-Y206C-Y206F-S210N) significantly correlate with HBsAg<1000IU/ml(*P*-value from <0.001 to 0.04). These mutations lie in divergent pathways involving other HBsAg C-terminus mutations: V190A + F220L (Phi = 0.41, *P* = 0.003), S204N + L205P (Phi = 0.36, *P* = 0.005), Y206F + S210R (Phi = 0.47, *P* < 0.001) and S210N + F220L (Phi = 0.40, *P* = 0.006). Notably, patients with these mutational pairs present HBsAg-levels 1log lower than patients without them(*P*-value from 0.003 to 0.02). *In-vitro*, the above-mentioned mutational pairs determined a significant decrease in HBsAg secretion-efficiency compared to wt(*P*-value from <0.001 to 0.02). Structurally, these mutational pairs reduced HBsAg C-terminus stability and determined a rearrangement of this domain.

In conclusion, HBsAg-levels in genotype-D are significantly lower than in genotype-A and -E in HBeAg-negative patients. In genotype-D, specific mutational clusters in HBsAg C-terminus correlate with lower HBsAg-levels *in-vivo*, hamper HBsAg-release *in-vitro* and affect its structural stability, supporting their detrimental role on HBsAg-secretion. In this light, genotypic-testing can be a valuable tool to optimize the clinical interpretation of HBsAg in genotype-D and to provide information on HBV-pathogenicity and disease-progression.

## Introduction

Worldwide, around 250 million individuals have a chronic hepatitis B virus (HBV) infection, and approximately 1 million individuals die yearly due to related illnesses [1]. Chronic HBV infection is a dynamic process reflecting a complex interplay between HBV and host immunity. During chronic HBV infection, the Hepatitis B “e” antigen (HBeAg) negative phase is associated with a large variety of clinical conditions ranging from HBeAg-negative infection (characterized by persistently serum HBV-DNA <2000IU/ml and normal ALT) to HBeAg-negative hepatitis where the interaction between the virus and the host immune cells leads to progressive liver necroinflammation, cirrhosis and hepatocellular carcinoma [2]. In clinical practice, HBsAg<1000IU/ml has been proposed to better define the status of HBeAg-negative infection in the setting of HBV genotype D [3]. However, a recent study has shown that the HBsAg levels can vary largely across the different HBV genotypes, with genotype D characterized by lower HBsAg levels than other genotypes, questioning the widespread application of this cut-off in the setting of non-D genotypes [4].

So far, ten HBV genotypes have been identified (A to J) showing a distinct geographic distribution, rate of disease progression and molecular differences in term of viral replication, viral protein expression and neutralization capacity [5–7]. At this regard, a previous study showed that HBV genotypes D and A were characterized by higher levels of HBV-DNA replication than genotypes B, C and J. Conversely, despite higher replicative potential, HBV genotype D showed the lowest production of HBeAg and HBsAg [6]. HBV genotypes also showed differential profiles of antibody binding, having potential implications for vaccine effectiveness [6].

The S gene is composed by pre-S1, pre-S2, and S region, coding for the three forms of HBV surface glycoproteins: the small-sized (hereafter defined as HBsAg), the medium-sized (pre-S2+S, M-HBsAg) and large-sized surface glycoprotein (pre-S1+pre-S2+S, L-HBsAg). These surface glycoproteins are synthesized in the endoplasmic reticulum (ER) where they rapidly undergo dimer and multimer formation via extensive disulphide bonding [8]. This results in budding into the ER as virions or as empty subviral particles, secreted with 100-fold to 100,000-fold higher levels than mature virions [9,10].

A recent study showed that in HBeAg-negative chronic HBV-infected patients, the ratio of the 3 HBV surface glycoproteins is strongly HBV genotype-dependent and can be also influenced by genetic variability in pre-S1 and pre-S2 regions [11].

Among the different HBsAg domains, the C-terminus (from amino acid [aa] 179 to 226) is hydrophobic and is inserted in the ER membrane [12,13]. This domain is involved in mediating the transit of the surface glycoproteins across the ER [12]. Mutations in this domain can result in a stable, glycosylated, but non-secreted protein, affecting the biogenesis and secretion of subviral particles [12]. Thus, it could be hypothesized that mutations in HBsAg C-terminus could affect HBsAg secretion, leading to lower HBsAg levels in clinical practise. So far, previous studies have investigated the impact of HBsAg mutations on HBsAg levels. However, most of them have focused on the so-called immune-escape mutations localized in the major hydrophilic region of HBsAg or on stop-codons that give origin to truncated forms of HBsAg that are retained in the endoplasmic reticulum and cannot efficiently secreted [14,15].

Based on this rationale, this study is aimed at evaluating (i) HBsAg levels in different HBV genotypes (D, A and E) in HBeAg-negative individuals, (ii) the correlation of mutations in the C-terminus HBsAg domain with lower HBsAg levels in HBeAg-negative individuals, infected with genotype D (iii) the impact of the identified mutations on HBsAg secretion in *in vitro* experiments and on HBsAg structural stability. To our knowledge this is one of the first studies aimed at investigating the impact of mutations in HBsAg C-terminus on HBsAg levels in clinical settings and in *in vitro* experiments.

## Materials and methods

### Study population

This study includes 323 consecutive treatment-naïve HBeAg-negative patients chronically infected with HBV, monitored for ≥1-year in different outpatients clinics in Italy, United Kingdom and Germany. For each patient (323/323), an available HBsAg sequence was obtained after ≥1-year monitoring. Among them, 136 had persistently serum HBV-DNA<2000 IU/ml and normal ALT, a profile compatible with HBeAg-negative infection [2].

Patients were excluded if co-infected with hepatitis C virus (HCV), hepatitis D virus (HDV) or human immunodeficiency virus (HIV).

Ethic approval was deemed unnecessary because, under Italian law, biomedical research is subjected to previous approval by ethics committees only in the hypothesis of clinical trials on medicinal products for clinical use (art. 6 and art. 9, leg. decree 211/2003). The research was conducted on viral DNA samples (used for clinical routine), and data previously anonymized, according to the requirements by Italian Data Protection Code (leg. decree 196/2003). Thus, all the plasma samples were already stored and not specifically collected for this study.

Data on demographics (sex, age, nationality), biochemistry (ALT, AST) and HBV serology and virology (serum HBsAg, HBV-DNA, HBV genotype), liver fibrosis assessment by liver biopsy or elastography were collected and stored in an *ad-hoc* designed anonymous database. Serum HBV-DNA was quantified using the COBAS AmpliPrep-COBAS TaqMan HBV test (Roche Diagnostics), with a lower limit of detection of 20 IU/ml and HBsAg was quantified using the Elecsys®HBsAgII assay (Roche Diagnostics), with a lower limit of detection of 0.05IU/ml.

### HBsAg population-based sequencing

HBsAg sequencing (1-226 aa) was performed on plasma samples, following a home-made protocol, as previously reported [16]. Further informations about the methodology are explained in the supplementary method (SM).

Phylogenetic analysis by the Tajima-Nei model (MEGA6.1) was performed to determine HBV genotype (details in SM).

### Association of HBV genotypes with HBsAg levels

Mann Whitney Test was applied to assess statistically significant differences in HBsAg levels in genotype D vs A and in genotype D vs E. Furthermore, a multivariate linear regression model was performed to evaluate potential association of genotype D, A, E with HBsAg levels after correction for gender, age, serum HBV-DNA, ALT and status of HBV infection.

The positive and negative predicted values of HBsAg<1000IU/ml to predict the status of HBeAg-negative infection were calculated according to the following assumptions:

- Positive predictive value (PPV) was defined as the probability that individuals with a HBsAg<1000IU/ml belong to the category of HBeAg-negative chronic infection,
- Negative predictive value (NPV) was defined as the probability that individuals without a HBsAg<1000IU/ml do not belong to the category of HBeAg-negative chronic infection.

Thus, to determine the PPV, the probability was calculated considering as numerator the number of patients with HBeAg-negative chronic infection and HBsAg<1000IU/ml (representing the true positives), and as denominator the overall number of patients with HBsAg<1000IU/ml (representing all positive tests). To determine the NPV, the probability was calculated considering as numerator the number of patients without HBeAg-negative chronic infection and HBsAg>1000IU/ml (representing the true negative tests), and as denominator the overall number of patients with HBsAg>1000IU/ml (representing all negative tests).

### Association of HBsAg C-terminus mutations with HBsAg levels

HBsAg sequences were used to assess the association of HBsAg C-terminus mutations with HBsAg<1000IU/ml. Mutations were defined according to the reference sequence of HBV-genotype D (reference sequence: X65259.1). The prevalence of HBsAg C-terminus mutations was calculated in 228 HBeAg-negative genotype D infected patients stratified according to HBsAg<1000IU/ml (*N* = 77) and HBsAg>1000IU/ml (*N* = 151). Statistically significant differences in the prevalence of mutations between the two groups of patients were assessed by the Fisher exact test. Benjamini-Hochberg method was used for multiple comparison corrections. Similarly, the association of HBsAg C-terminus mutations with HBsAg<1000IU/ml was carried out also in the subset of 91 patients with HBeAg-negative chronic infection sustained by genotype D: 52 with HBsAg<1000IU/ml and 39 with HBsAg>1000IU/ml. The prevalence of mutations was calculated in these two groups of patients and compared to verify statistically significant differences by Fisher Exact test. This analysis was carried out in order to further corroborate results in two subgroups of patients with comparable serum HBV-DNA levels, thus limiting the impact of serum HBV-DNA on the observed associations.

### Co-variation analysis among mutations and its correlation with levels of serum HBsAg and HBV-DNA

In the set of 228 HBeAg-negative genotype D infected patients, the patterns of pairwise interactions among aa substitutions in HBsAg C-terminus were analysed (details in SM) [17].

Furthermore, in the overall population of 228 HBV genotype-D infected patients, the median (IQR) levels of serum HBsAg and HBV-DNA was determined for each mutation identified (single or in pair). These levels were then compared to those observed in the set of patients without evidence of any of these identified mutations (arbitrarily defined as *wt*) in order to define statistically significant differences by Mann–Whitney test.

### Impact of mutations in HBsAg C-terminus on HBsAg quantification

In order to investigate the impact of the mutations (single or in pairs) identified in this study on HBsAg secretion, a plasmid encoding the small HBsAg linked to a streptavidin-tag version II at N-terminus (Strep-Tactin coated microplate, IBA Lifescience, Germany, strep-tag) was used to transfect the HepG2 cells. Further information about the methodology are explained in SM.

HepG2 cells were grown in a 37°C humidified atmosphere containing 5% CO2, using Dulbecco’s modified Eagle’s medium (DMEM) (Life Technologies, Inc., Gaithersburg, MD) supplemented with 10% fetal bovine heat-inactivated serum and with 100 U/ml penicillin + 100 µg/ml streptomycin + 2 mM L-glutamine.

2 μg of *wt* and mutated plasmids were transfected into HepG2 cells using the TransIT-X2 Transfection Reagent (Mirus Bio LLC, USA), according to manufacturer’s instructions. All transfection experiments were performed in 6 wells plates and, for each well, 500,000 cells in 2 ml of medium were seeded.

Furthermore, all transfections included 0.1 μg of green fluorescence protein expression vector (GFP) to assess transfection efficiency. Both cell fractions and culture supernatants were harvested at 72 hours post-transfection. For each mutant, at least 3 independent transfection experiments were performed each led in duplicate.

The amount of strep-tagged HBsAg released in culture supernatants was quantified using a specifically-designed ELISA capable to recognize the Strep-tag linked to the HBsAg (defined hereafter as Strep-tag ELISA). Differently from the commonly used HBsAg assays, the Strep-tag based ELISA is not influenced by HBsAg mutations, giving the advantage to discriminate between a reduction of HBsAg recognition (due to an impaired anti-HBs binding) and a decrease in HBsAg production and secretion.

Moreover, the amount of extracellular and intracellular strep-tagged HBsAg was measured by a commercially available ELISA (LIAISON® XL murex HBsAg Quant, DiaSorin, Italy), and used to calculate the HBsAg secretion factor defined as the ratio of extracellular HBsAg on intracellular HBsAg. A reduction of HBsAg secretion factor compared to *wt* reflects a defect on HBsAg secretion in presence of the mutation(s). Differently from the other commercially available assays, the LIAISON® XL murex HBsAg Quant assay targets multiple epitopes along the entire HBsAg proteins, thus limiting the impact of mutations, capable to affect HBsAg antigenicity, on the quantification of HBsAg. For this reason, this assay was used to further corroborate the impact of mutations on HBsAg secretion.

In detail, the plasmid encoding for Strep-tagged HBsAg was constructed as previously described [16]. The following mutations were introduced: V190A, V190A + F220L, S204N, S204N + L205P, Y206F, Y206F + S210R, Y206F + V194A, Y206F + S204T, Y206F + M197T, S210N, S210N + F220L.

### In silico prediction of HBsAg three-dimensional structure

I-TASSER (Iterative Threading ASSEmbly Refinement) was used to generate the 3D structure of HBsAg starting from its amino acid sequence following an homology modelling approach. The accuracy of structure prediction was defined according to the confidence score defined as C-score [18,19]. I-Tasser is a widely used approach ranked as the top method for the prediction of the 3D structure of proteins. The robustness of this approach has been reported in different manuscripts published on leading journals [19–21].

The fold-stability change (ΔΔG) between *wt* and mutants was calculated by STRUM, with ΔΔG[*wt*-mutated]<0 indicating a reduced stability in presence of the mutations [22] (Details in SM).

## Results

### Patients’characteristics

This study includes 323 HBeAg-negative patients naive to anti-HBV drugs and monitored for at least 1 year, each with an available HBsAg sequence. Most patients were male with a median age of 40 (32–53) years (Table 1). Median (IQR) serum HBV-DNA and HBsAg were 3.0 (2.9–4.3) logIU/ml and 3.5 (2.9–3.9) logIU/ml, while median (IQR) ALT and AST were 31 (23–47) U/L and 24 (18–33) U/L, respectively (Table 1). By phylogenetic analysis, 228 patients were infected with HBV genotype D, 65 with genotype A and 30 with genotype E (Table 1). Median (IQR) serum HBV-DNA was comparable among HBV genotypes: 3.6(2.9–4.4) logIU/ml for genotype D, 3.5(2.9–4.4) logIU/ml for genotype A and 3.2(2.5–3.6) logIU/ml for genotype E.

**Table 1.** Patients’ characteristics.

Patients’ characteristics	Overall (*N* = 323)	Genotype D (*N* = 228)	Genotype A (*N* = 65)	Genotype E (*N* = 30)
Male, *N* (%)	197 (61.0)	135 (59.2)	32 (49.2)	30 (100)
Median age, years (IQR)	40 (32-53)	42 (35-55)	42 (31-48)	27 (25-31)
***Nationality:***				
Eastern European, *N* (%)	96 (29.7)	77 (33.8)	19 (29.2)	2 (6.7)
Northern European, *N* (%)	78 (24.2)	40 (20.6)	29 (44.6)	0 (0)
Southern European, *N* (%)	85 (26.3)	83 (36.4)	2 (3.1)	0 (0)
African, *N* (%)	43 (13.3)	3 (1.3)	12 (18.5)	28 (93.3)
Asian, *N* (%)	21 (6.5)	18 (7.9)	3 (4.6)	0 (0)
***HBV characteristics:***				
HBeAg negative status, *N* (%)	323 (100)	228 (100)	65 (100)	30 (100)
Median (IQR) HBV-DNA, log IU/ml	3.0 (2.9-4.3)	3.6 (2.9-4.5)	3.5 (2.9-4.4)	3.2 (2.5-3.7)
Median (IQR) ALT, IU/ml	31 (23-47)	31 (23-46)	31 (26-47)	27 (21-55)
Median (IQR) AST, IU/ml	24 (18-33)	23 (17-33)	25 (18-36)	25 (21-32)

Abbreviations: ALT, alanine aminotransferase; AST, aspartate aminotransferase; HBsAg, hepatitis B surface antigen.

Among 323 HBeAg-negative patients, 136 had persistently serum HBV-DNA<2000IU/ml and normal ALT during the >1-year monitoring before the collection of blood sample for this study, a profile compatible with HBeAg-negative infection [2]: 91 with genotype D, 29 with genotype A and 16 with genotype E. Among these 136 patients, liver fibrosis assessment was available for 32 patients: all of them were characterized by with no/limited fibrosis.

Furthermore, 105 belonged to the grey-zone category with a serum HBV-DNA ranging from 2000IU/ml to 20,000IU/ml. Among them, liver fibrosis assessment was available for 31 patients. Most of them had no/limited fibrosis (*N* = 27), the remaining 4 had moderate up to severe fibrosis.

The remaining 82 patients had a serum HBV-DNA >20,000IU/ml. Most of them (*N* = 58) had ALT persistently >40U/L and the remaining 24 had fluctuating ALT during the >1-year monitoring before blood sample collection for this study, all showing a profile compatible with HBeAg-negative chronic hepatitis. Liver fibrosis assessment was available for 36 patients. Most of them (*N* = 24) had moderate/severe fibrosis. In particular, 10 patients had cirrhosis.

In our study population, 98 patients met criteria for eligibility to treatment according to EASL guidelines (82 patients with HBeAg-negative chronic hepatitis and 16 in the grey zone category) [2].

#### HBsAg levels according to HBV genotypes

**Figure 1.**
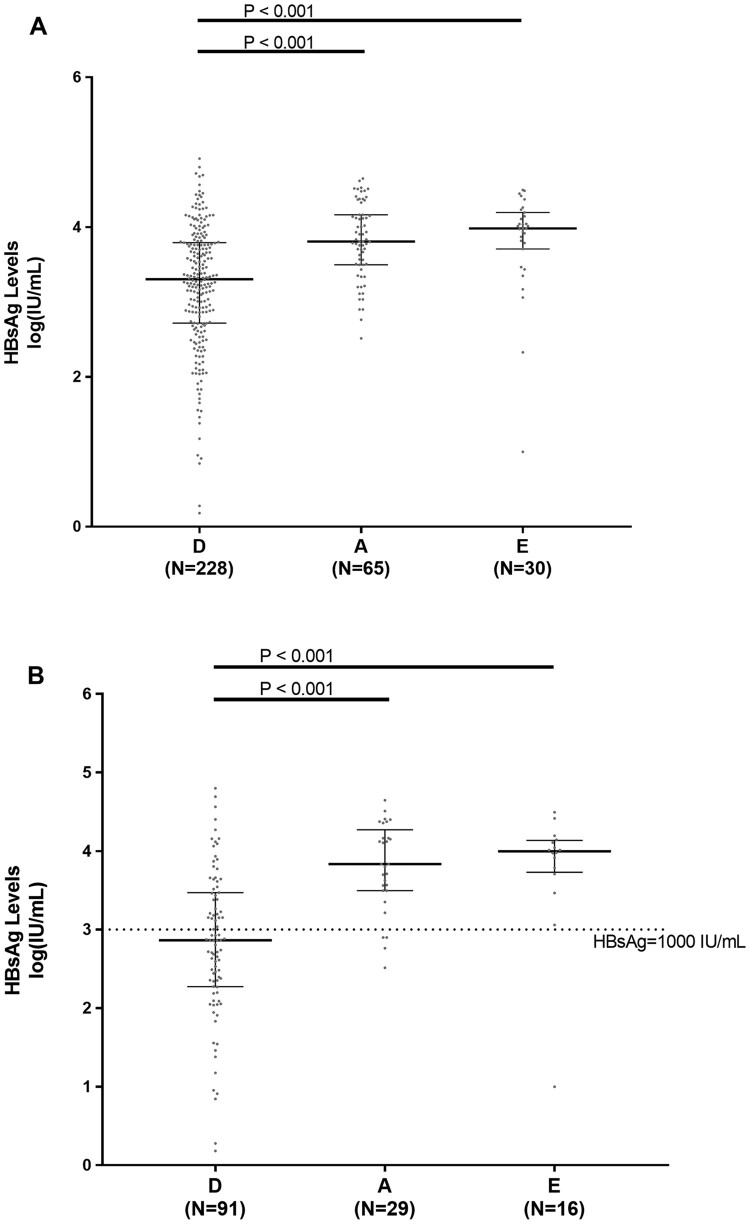
HBsAg levels in HBV genotypes D, A and E. Scatter plots report the distribution of HBsAg levels in the overall population of HBeAg-negative patients (N=228 for genotype D, N=65 for A and N=30 for E) (A) and in patients with persistent serum HBV-DNA<2,000IU/ml and normal ALT (N=91 for D, N=29 for A and N=16 for E) (B). Statistically significant differences were assessed by Mann-Whitney test. Bars represent median, 25^th^ and 75^th^ percentile.

Among the 323 HBeAg-negative patients, HBsAg levels differed significantly across HBV genotype D, A and E. In particular, HBV genotype D was characterized by significantly lower HBsAg levels than HBV genotype A and E (3.3[2.7–3.8]IU/ml vs 3.8[3.5–4.2]IU/ml and 3.9[3.7–4.2]IU/ml, *P*<0.001 for D versus A and *P*<0.001 for D versus E) (Figure 1(A)). This result was confirmed when the analysis was focused on 136 patients with serum HBV-DNA <2000IU/ml and persistently normal ALT: 2.7[2.3–3.5]IU/ml for genotype D vs 3.8[3.5–4.3]IU/ml for genotype A and 4.0[3.7–4.1]IU/ml for genotype E (*P* < 0.001 for D versus A and *P* < 0.001 for D versus E) (Figure 1(B)).

The positive and negative predictive value of HBsAg<1000IU/ml in predicting HBeAg-negative infection were 67.5% and 74.2% for genotype D, 44.4% and 55.4% for genotype A, and 50% and 46.4% for genotype E.

Multivariate linear regression model confirms that HBV genotype D was associated with HBsAg levels lower than genotype A (*P* < 0.001) and E (*P* = 0.022) after correction for patients’ demographics, serum HBV-DNA, ALT and status of HBV infection (Table S1). As expected, age and serum HBV-DNA were other independent factors negatively and positively associated with the amount of HBsAg levels (*P* < 0.0001 and *P* = 0.0016), respectively (Table S1).

### HBsAg C-terminus mutations underlying lower HBsAg levels in HBeAg-negative individuals infected with HBV genotype D

In order to unravel factors contributing to lower HBsAg levels in HBV genotype D, the attention was focused on HBsAg C-terminus since it plays an important role in secretion of HBV surface glycoproteins [12]. Among 228 patients infected with genotype D, HBsAg C-terminus genetic variability resulted significantly higher in patients with HBsAg<1000IU/ml (*N* = 77) than in patients with HBsAg>1000IU/ml (*N* = 151) (mean[ ± ] genetic distance:41 ± 24 substitutions/1000 nucleotides vs 22 ± 17 substitutions/1000 nucleotides, *P* < 0.001). This suggests a potential impact of the genetic variability in this region on HBsAg secretion, thus prompting us to investigate the existence of specific mutations in HBsAg C-terminus specifying lower HBsAg levels in HBV genotype D.

**Figure 2.**
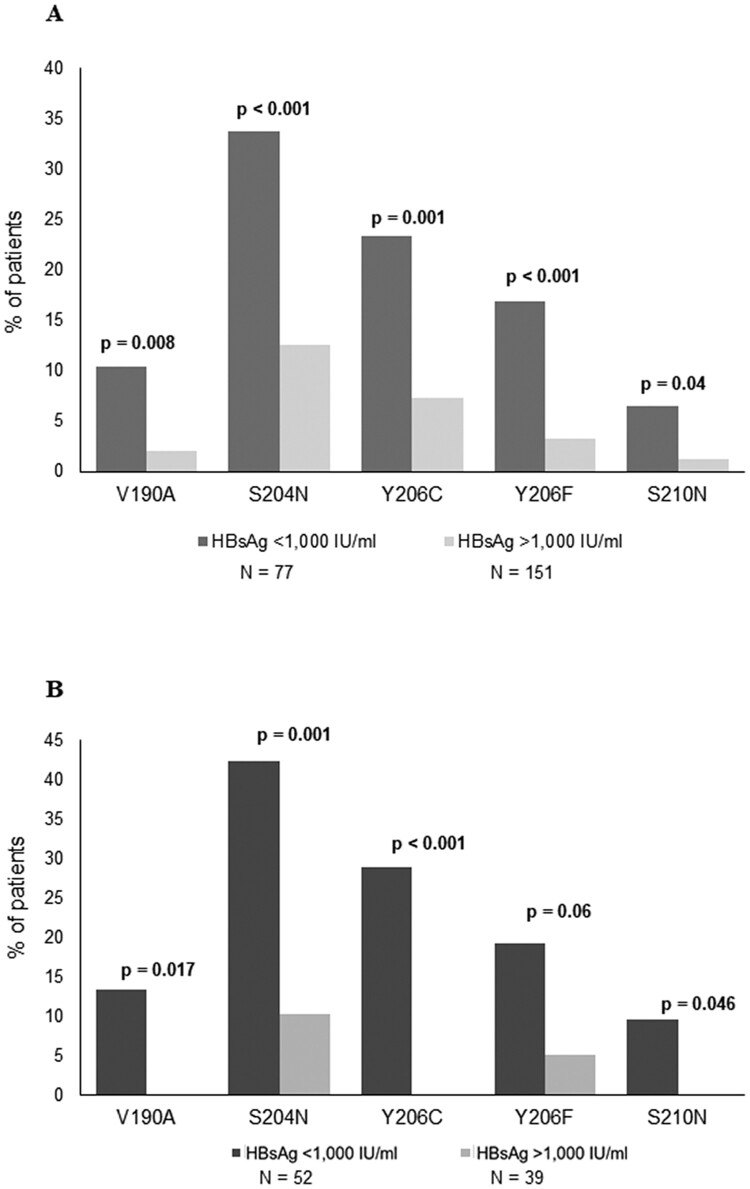
Mutations in HBsAg C-terminus associated with HBsAg<1,000IU/ml. The prevalence of each mutation in HBsAg C-terminus (amino acids: 170-226) was calculated in 228 HBV genotype-D infected patients stratified according to HBsAg levels (N=77 with HBsAg <1,000IU/ml and N=151 with HBsAg >1,000IU/ml) (A) and in 91 patients with HBeAg-negative genotype-D infection (N=52 with HBsAg<1,000IU/ml and N=39 with HBsAg>1,000IU/ml) (B). Statistically significant differences were assessed by Fisher Exact Test. In (A) Benjamini-Hochberg Method was used for multiple comparison correction. Statistically significant differences were confirmed after correction for multiple comparison for all mutations with the exception of S210N.

By stratifying 228 HBeAg-negative genotype D infected patients according to HBsAg levels, 5 specific mutations (V190A, S204N, Y206C, Y206F and S210N) resulted significantly correlated with HBsAg<1000IU/ml (Figure 2). Their prevalence ranged from 6.5% to 33.7% in patients with HBsAg<1000IU/ml versus 1.3%–12.6% in patients with HBsAg>1000IU/ml (Figure 2(A)). Overall, 58.4% of patients with HBsAg<1000IU/ml carried at least one of these mutations. These results were confirmed also restricting the analysis on the subgroup of patients with HBeAg-negative infection (serum HBV-DNA<2000IU/ml and persistently normal ALT) (*N* = 91, Figure 2(B)) stratified in HBsAg<1000IU/ml (*N* = 52) and HBsAg>1000IU/ml (*N* = 39). In particular, the mutations had a prevalence ranging from 9.6% to 42.3% in patients with HBsAg<1000IU/ml and from 0 to 10.3% in patients with HBsAg>1000IU/ml (Figure 2(B)).

Interestingly, among the above-mentioned HBsAg mutations, S204N was the only detected with higher prevalence in patients with HBsAg<1000IU/ml than in those with HBsAg>1000IU/ml in HBV genotype A (100% for HBsAg<1000IU/ml versus 12.9% for HBsAg>1000IU/ml, *P* = 0.003) (Table S4, Fig. S2). Similarly, considering the overall population of patients infected with HBV-genotype E, 6 patients were characterized by the presence of S204N (Table S4, Fig. S2). Interestingly, in these patients, HBsAg levels tend to be lower than in those without this mutation (7835[6400–9776]IU/ml versus 10568[7097–16032]IU/ml), even if this difference is not statistically significant.

### Association among HBsAg C-terminus mutations

The next step of our study was to investigate the association of the mutations identified in the previous paragraph with any other HBsAg C-terminus mutation. All the mutations associated with HBsAg<1000IU/ml (with the exception of Y206C) were found significantly correlated in pairs with other HBsAg C-terminus mutations. In particular, V190A and S210N were both specifically correlated with F220L (Phi = 0.30, *P* = 0.003 for both), while S204N showed positive correlation with L205P (Phi = 0.25) (Table 2). Y206F showed multiple positive correlations with different HBsAg C-terminus mutations: V194A (phi = 0.23), M197T (phi = 0.32), S204T (phi = 0.26) and S210R (phi = 0.33) (Table 2). Notably, these pairs of mutations were never found or occurred only in 1 out of 151 patients with HBsAg>1000IU/ml (prevalence: 0.7%) (Table 2).

**Table 2.** Association between HBsAg C-terminus mutations correlated with HBsAg <1000 IU/mL and other HBsAg C-terminus mutations.

Mutations associated with HBsAg<1000 IU/ml	Correlated mutations	Phi^a^	*P*-Value^a^	*N* (%) of the pairs of HBsAg mutations	
				<1000 IU/ml^b^	>1000 IU/ml^b^
**V190A**	**F220L**	0.30	0.003	5 (6.5)	0
**Y206F**	**V194A**	0.23	0.009	4 (5.2)	1 (0.7)
** **	**M197T**	0.32	0.002	4 (5.2)	0
** **	**S204T**	0.26	0.006	4 (5.2)	0
** **	**S210R**	0.33	<0.001	7 (9.1)	1 (0.7)
**S204N**	**L205P**	0.25	0.004	4 (5.2)	1 (0.7)
**S210N**	**F220L**	0.30	0.003	4 (5.2)	0

^a^Binomial correlation coefficient (Phi) was calculated to assess the strength of association for each pair of identified mutations. Statistically significant differences were assessed by Fisher Exact Test.

^b^The prevalence of the pairs of mutations was calculated in the group of 77 patients with HBsAg <1,000IU/ml and 151 patients with HBsAg >1,000IU/ml. All the differences in the prevalence were statistically significant (*P*<0.05) by Fisher exact test.

By multivariable analysis, the presence of ≥1 of these pairs of mutations was independently associated with HBsAg<1000IU/ml after correction for patient’s demographics, serum HBV-DNA and ALT (O.R.[95%CI]:14.75[1.83–118.79], *P* = 0.011) (Table 3). Patient’s age and serum HBV-DNA were other independent factors showing a positive and negative association with HBsAg<1000IU/ml, respectively (O.R.[95%CI]:1.04[1.02–1.07], *P* = 0.002 for age and O.R.[95%CI]:0.46[0.33–0.63], *P* < 0.001 for serum HBV-DNA) (Table 3).

**Table 3.** Factors associated with HBsAg levels <1000 IU/ml in HBeAg-negative patients infected with HBV genotype D.

Variables	Univariate analysis^a^		Multivariate analysis^a^	
	Crude OR [95% CI]	*p*-value	Adjusted OR [95% CI]	*p*-value
Gender (Female vs. Male^b^)	1.21 (0.69-2.14)	0.49		
Age (for 1 year increase)	1.04 (1.02-1.06)	<0.001	1.04 (1.02-1.07)	0.002
HBV-DNA, log_10_ IU/ml	0.41 (0.30-0.56)	<0.001	0.46 (0.33-0.63)	<0.001
ALT, U/L	1.00 (0.99-1.00)	0.143		
≥1 pair of mutations	39.34 (5.11-303.22)	<0.001	14.75 (1.83-118.79)	0.011

^a^Univariate and multivariate logistic regression analysis was performed on 228 HBeAg-negative patients infected with HBV genotype D. The following variables were considered: gender, age, HBV-DNA log10, ALT, >1 pair of mutations associated with HBsAg<1000 IU/ml. Variables with *P*-value<0.05 in univariate analysis were included in multivariate analysis.

^b^Reference group

Results confirmed also in the subset of 91 patients with HBeAg negative genotype D infection (Table S2). Overall findings support the existence of divergent pathways underlying lower HBsAg levels in HBV genotype D.

Lastly, the pairs of HBsAg mutations, associated with HBsAg<1000 IU/ml, correspond to ≥1 mutation in RT, all residing in the spacer between the RT C and D domains, not known to have any impact on RT activity (Table S3).

Furthermore, the presence of nonsense mutations, giving origin to stop-codons (known to affect HBsAg secretion) was also investigated. Stop-codons were detected in 14/228 HBeAg-negative genotype D infected patients (6.1%) with median(IQR) HBsAg levels of 3.4(2.8–4.0)IU/ml. By covariation analysis, no statistically significant association was found between the presence of stop-codons and the pairs of mutations associated with HBsAg<1000IU/ml (phi = 0.10, *P* = 0.126), suggesting that they lay on divergent evolutionary pathways.

### Correlation of mutational patterns in HBsAg C-terminus with serum HBsAg and HBV-DNA levels in vivo

In 228 HBeAg-negative genotype D infected patients, the median(IQR) HBsAg levels in presence or in absence of the above-mentioned pairs of mutations were determined in order to corroborate their association with lower HBsAg levels.

**Figure 3.**
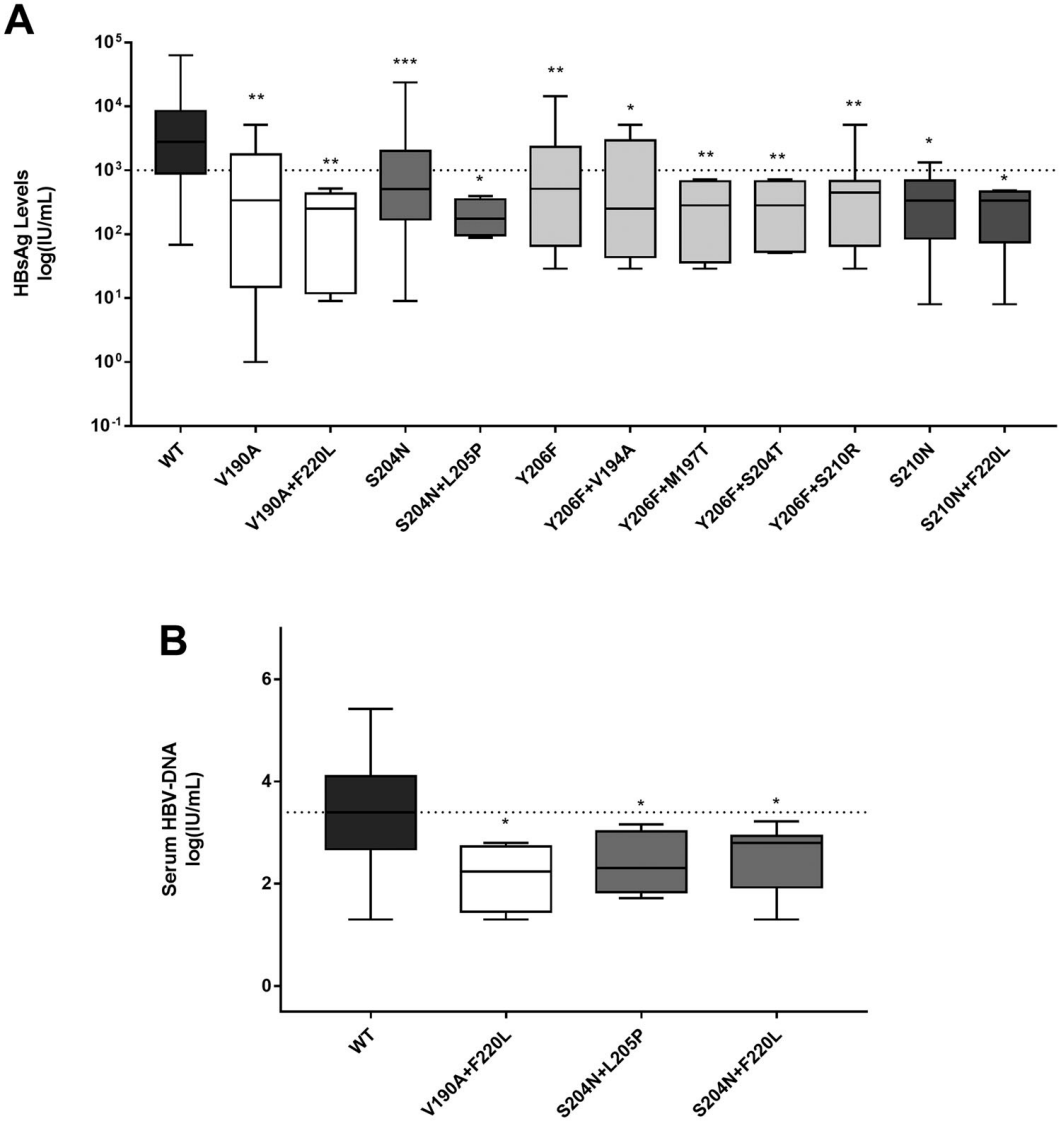
Serum HBsAg and HBV-DNA in presence of HBsAg C-terminus mutations associated with HBsAg<1,000IU/ml. Box plots report the distribution of serum HBsAg (A) and HBV-DNA (B) observed in presence of mutations (single or in pair) associated with HBsAg<1,000IU/ml in 228 HBeAg-negative genotype D infected patients. Statistically significant differences were assessed by Mann-Whitney Test. Wild-type (*wt*) indicates a virus without any mutation associated with HBsAg<1,000IU/ml. *indicates a *P* value ranging from 0.05 to 0.01, **indicates a *P* value from 0.001 to 0.01, and ***indicates *P*<0.001.

The pairs of mutations identified in previous paragraph were significantly associated with median (IQR) HBsAg levels lower (at least of 1log) than those observed for *wt* (Figure 3(A)). Furthermore, some pairs of mutations (V190A + F220L, S204N + L205P, S204N + F220L) were also associated with lower median (IQR) serum HBV-DNA (Figure 3(B)). The presence of the single or pairs of mutations remained significantly associated with lower HBsAg levels also in the subset of 91 patients with HBeAg-negative genotype D infection (data not shown).

### Impact of HBsAg C-terminus mutations on HBsAg secretion in in vitro experiments

The impact of HBsAg C-terminus mutations on HBsAg secretion was evaluated in *in vitro* experiments. For this purpose, an assay *ad hoc* designed to identify defects in HBsAg secretion was used (see methods section). The single mutation S204N and S210N determined a 31% and 23% decrease in HBsAg release compared to *wt* (*P* = 0.004 and *P* = 0.001, respectively).

**Figure 4.**
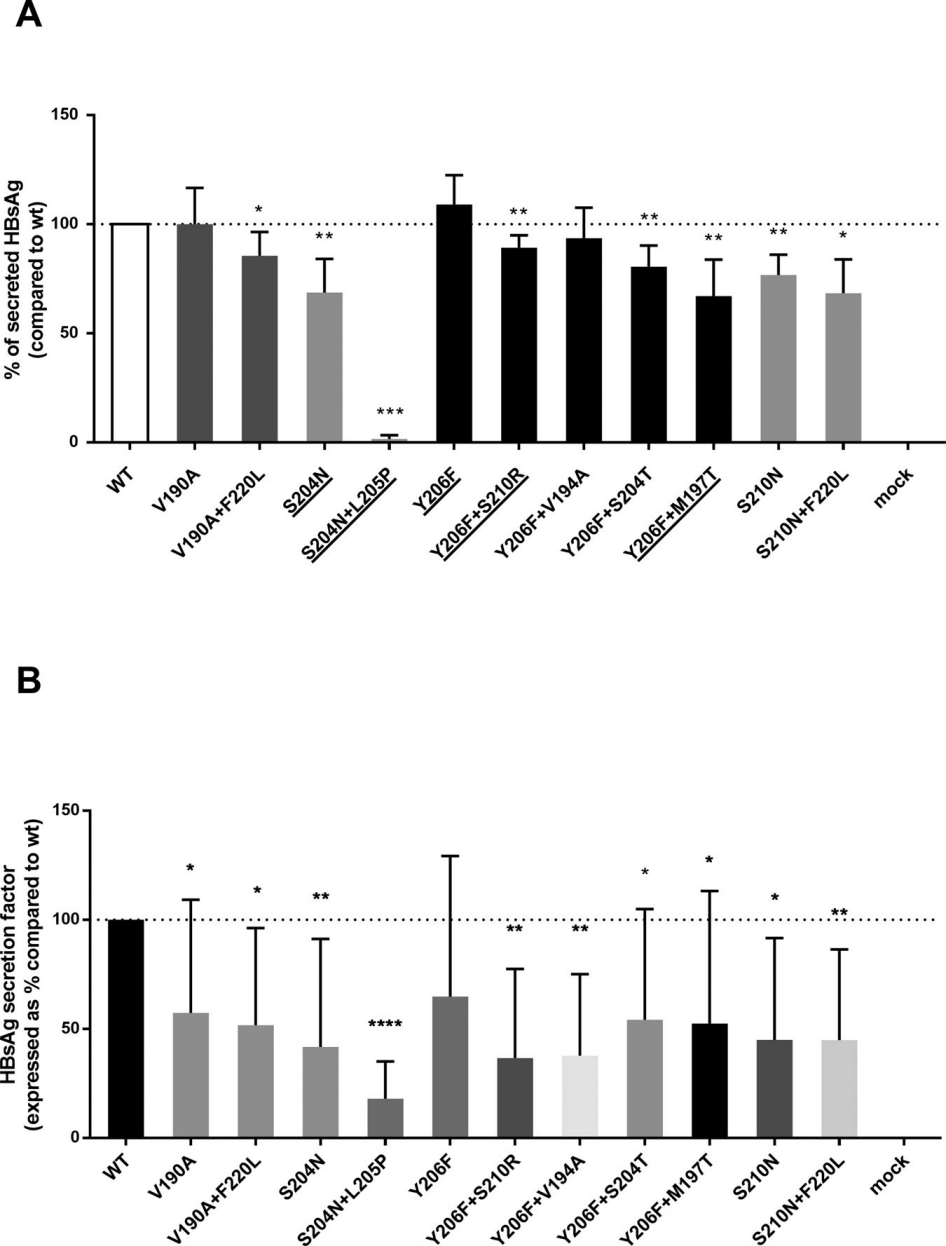
*In vitro* impact of HBsAg C-terminus mutations on HBsAg quantification. A plasmid encoding the small HBsAg linked to a streptavidin-tag version II at N-terminus was used to transfect the HepG2 cells. (A) The amount of strep-tagged HBsAg released in culture supernatants was then quantified using a specifically-designed ELISA capable to recognize the Strep-tag linked to the HBsAg. For each mutant, the amount of strep-tagged HBsAg released in supernatants of HepG2 cell cultures was expressed as percentage, considering the amount of the wild-type strep-tagged HBsAg as 100%. Results represent the mean values (+/- standard deviation) of 3 independent experiments, each led in duplicate. * indicates *P* values ranging from 0.05 to 0.01, ** *P* values from 0.01 to 0.001 and *** *P* values <0.001 compared to wild-type. For the underlined pairs of mutations HBsAg release was significant also compared to the corresponding single mutation S204N (*P*<0.001) or Y206F (*P*=0.007, *P*=0.001 and <0.0001, respectively). (B) For each mutant, the HBsAg secretion factor was measured as the ratio between extracellular and intracellular strep-tagged HBsAg in HepG2 cells. The amount of extracellular and intracellular strep-tagged HBsAg was measured by the LIAISON® XL murex HBsAg Quant assay (DiaSorin, Italy). HBsAg secretion factor of mutants was expressed as percentage, considering the amount of the wild-type strep-tagged HBsAg as 100%. Results represent the mean values (+/- standard deviation) of 3 independent experiments, each led in duplicate. * indicates *P* values ranging from 0.05 to 0.01, ** *P* values from 0.01 to 0.001, *** *P* values from 0.001 to 0.0001 and **** *P* values <0.0001 compared to wild-type.

All the pairs of mutations (with the exception of Y206F + V194A) significantly decreased the amount of extracellular HBsAg compared to *wt* (*P* values ranging from 0.022 to <0.001) paralleling their association with lower HBsAg levels *in vivo* (Figure 4(A)). For the pair of mutations S204N + L205P, a 99% decrease was observed (Figure 4(A)). Notably, for the mutational pairs S204N + L205P, Y206F + S210R, Y206F + S204T and Y206F + M197T, the decrease in HBsAg release was significant also compared to the corresponding single mutation S204N (*P* < 0.001) or Y206F (*P* = 0.007, *P* = 0.001 and <0.0001, respectively) (Figure 4(A)). Superimposable results were obtained when extracellular HBsAg was quantified by using a commercially available assay, directly targeting HBsAg (data not shown).

In line with these data, all the above-mentioned mutations were also associated with a decrease in HBsAg secretion factor compared to *wt* (P from <0.0001 to 0.05), supporting their detrimental effect on HBsAg secretion (Figure 4(B)). Again, the strongest decrease (91%) in HBsAg secretion factor was observed for the pair S204N + L205P (Figure 4(B)).

### Structural analysis

**Figure 5.**
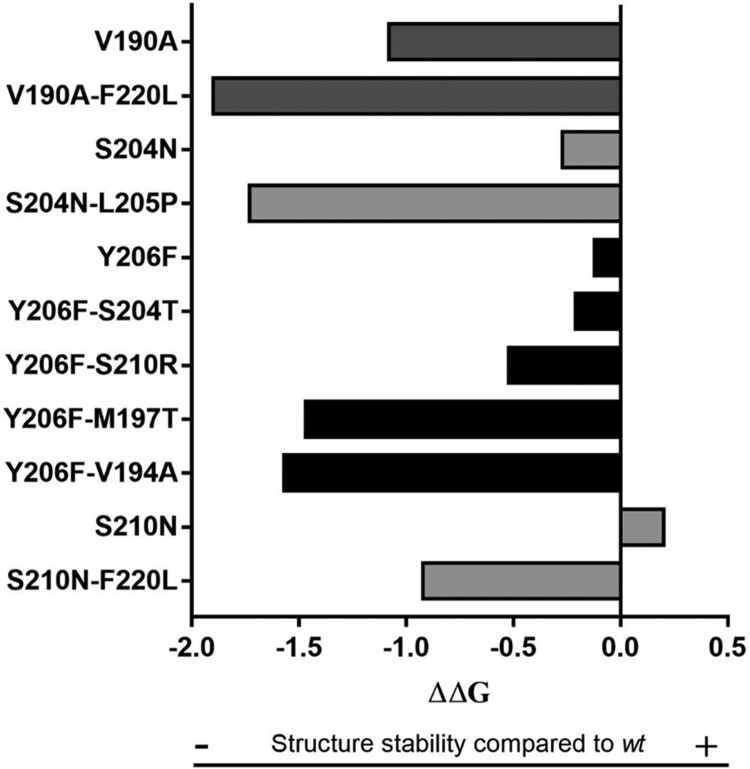
HBsAg structure stability in presence of C-terminus mutations associated with HBsAg <1,000IU/ml. The histogram shows the variation of HBsAg C-terminus stability in presence of mutations (single or in pairs) associated with lower HBsAg levels. HBsAg C-terminus stability was measured by calculating ΔΔG by STRUM *(*Quan et al., Bioinformatics, 2016). ΔΔG [wt-mutated] < 0 indicates a reduced stability in presence of the mutations.

HBsAg C-terminus is composed by two transmembrane α-helices spanning aa 188–198 and 205–226 linked by a cytosolic loop (Fig. S1). A final step of our study was to investigate the impact of the identified mutational profiles on the stability and structural conformation of HBsAg C-terminus. The pairs of mutations determined a decrease in HBsAg stability even higher than that observed for the single mutations (Figure 5).

The decrease in HBsAg stability can be explained by the fact that the pairs of mutations alter the structural conformation of C-terminus domain by reducing the length of the fourth transmembrane HBsAg domain (Table 4).

**Table 4.** Structural modifications in the length of the 4th α-helix induced by HBsAg C-terminus mutations associated with HBsAg<1000 IU/ml.

Mutations in HBsAg C-terminus	Length of the 4th alpha helix^a^	
	Residues	N° of residues
**Wild Type**	**L205-Y225**	**21**
**V190A**	L209-Y225	17
**V190A + F220L**	L205-V224	20
**S204N**	L209-Y225	17
**S204N + F220L**	L209-Y225	17
**S204N + L205P**	L209-Y225	17
**Y206F**	F212-Y225	14
**Y206F + S204T**	L209-Y225	17
**Y206F + S207I**	L209-Y225	17
**Y206F + S210R**	L205-V224	16
**Y206F + V194A**	F206-V224	19
**Y206F + M197T**	L206-Y225	20
**S210N**	G202-V224	23
**S210N + F220L**	S204-Y225	22

^a^The 3D-structures of *wt* and mutated HBsAg were predicted by a homology modeling approach using a reference validated HBsAg structure and elaborated by I-TASSER server.

## Discussion

This study shows that HBV genotype D is associated with HBsAg levels lower than genotype A and E in HBeAg-negative patients. This result was confirmed by multivariable analysis and in the subgroup of patients with persistently serum HBV-DNA<2000IU/ml and normal ALT. Our results are in line with recent *in vitro* studies showing that HBV genotype D, despite showing a higher efficiency in replication, was characterized by a lower HBsAg release than the other HBV genotypes, particularly HBV genotype A [6,23]. Furthermore, in agreement with a recent study [24], we found that HBV genotypes have an impact on HBsAg amount without affecting serum HBV-DNA. This finding that can be explained by the fact that serum HBV-DNA corresponds to the relaxed circular DNA, that is produced by the reverse transcriptase using the pre-genomic RNA as template, and then incorporated into virions. Conversely, HBV surface glycoproteins are produced from sub-genomic mRNAs and integrated DNA [25] and are incorporated into virions and, in large amount, in subviral particles. Different HBsAg levels among HBV genotypes despite comparable serum HBV-DNA can be explained by factors that alter the release of subviral particles without affecting the release of virions.

Overall findings reinforce the need to identify genotype-specific HBsAg thresholds for a better staging of patients with chronic HBeAg-negative infection. This is critical considering that HBsAg quantification can be a valuable tool for the differential diagnosis between HBeAg-negative infection and HBeAg-negative hepatitis, can help in individualizing the frequency of follow-up and in identifying patients more prone to achieve HBsAg loss in the setting of HBeAg-negative infection [26].

In order to unravel additional factors underlying lower HBsAg levels in genotype D, the attention was focused on the extent of genetic variability in HBsAg C-terminus, known to be important for secretion of HBV surface glycoproteins [12].

In particular, in the setting of HBeAg-negative genotype D infection, we identified specific mutations (single or in pairs) significantly correlated with lower HBsAg levels. Some pairs of the identified mutations reduce HBsAg secretion efficiency and HBsAg stability by shortening the the 4th transmembrane C-terminus domain. Notably, for the pair of mutation S204N + L205P a 90% decrease in HBsAg secretion efficiency was observed. It is noteworthy to mention that this pair of mutations has been detected exclusively in patients infected with genotype D, and never in genotype A and E, suggesting a potential interference of the genetic backbone on the development of this mutational pattern.

It is conceivable that the mutational patterns identified in this study can alter the correct HBsAg folding in the ER membrane, thus affecting the trafficking of HBsAg across the ER, and in turn its extracellular secretion. Interestingly, it is known that the accumulation of misfolded proteins in the ER can lead to the activation of a finely regulated cellular program defined as “unfolded protein response”. This program induces the degradation of ER via autophagy in order to prevent the activation of cell death pathways [27]. Notably, a previous study showed that HBsAg by itself can trigger autophagy, and the activation of the autophagic process can contribute to viral replication [28,29]. In this light, by activating autophagy, the identified mutations might favour the persistence of intrahepatic viral reservoir explaining their selection in viral quasispecies.

Furthermore, it has been demonstrated that defects in HBsAg secretion have been associated with an accumulation of cccDNA in the nuclei, suggesting a role of the identified mutations in modulating cccDNA synthesis and amplification [30].

In our study, results obtained *in vitro* experiments usually parallel those observed in patients and by structural analysis. A discrepancy is observed for S210N and S210N + F220L. Indeed, in *in vitro* experiments, both S210N and S210N + F220L determined a significant decrease in HBsAg amount compared to wild-type. Nevertheless, the decrease observed for S210N + F220L was not significantly stronger than that observed for S210N, paralleling results observed in patients. Conversely, structural analysis showed a decreased stability only for the double mutant. It is conceivable to hypothesize that, beyond structural stability and folding in the membrane of endoplasmic reticulum, cellular factors (that cannot be considered in the structural analysis) can be implicated in mechanisms underlying HBsAg release.

Thus, in presence of these mutational patterns, the detection of a low HBsAg titer can be ascribable to an impaired HBsAg secretion and not to a limited intrahepatic HBV reservoir, thus potentially leading to a misinterpretation of HBsAg levels in the clinical settings in which HBsAg quantification is applied. Overall findings support the use of genotypic testing for an optimized interpretation of HBsAg measurements particularly in the setting of HBeAg-negative HBV infection sustained by genotype D. Furthermore, these results reinforce the use of genotypic testing to provide information on mutational profiles that can affect HBV-induced pathogenesis and in turn disease progression.

In this study, we have sequenced HBV genome extracted from viral particles circulating in the blood stream of infected patients. This supports that the HBsAg mutational profiles, analysed in this study, are present in the relaxed circular HBV-DNA incorporated into virions deriving from cccDNA (after the retro-transcription of pre-genomic RNA). Nevertheless, there is increasing evidence that in the setting of HBeAg-negativity, HBsAg can also derive from integrated HBV-DNA [11,25,31–33]. Further studies are necessary to unravel the existence of a potential genetic compartmentalization between cccDNA and integrated HBV-DNA and its impact on HBV pathogenicity and immunogenicity.

Finally, this study has some potential limitations. In particular, this study has analysed HBV genotypes D and A (the most common in Europe) and also HBV genotype E imported from West Africa. Further studies are necessary to investigate the levels of HBsAg in the other HBV genotypes. Furthermore, HBsAg sequences were obtained by a population-based sequencing approach that does not allow to provide the burden of a given mutation in the viral quasispecies. Lastly phi coefficients, reflecting the strength of association between two mutations, although being all statistical significant, never exceeded 0.30, suggesting the need of further studies with larger sample size to unravel the evolutionary pathways leading to mutations coevolution.

In conclusion, HBsAg levels in HBV genotype D are significantly lower than in genotype A and E in the setting of HBeAg-negative chronic infection. In genotype D infected patients, specific clusters of mutations in HBsAg C-terminus correlate with lower HBsAg levels *in vivo*, hamper HBsAg release *in vitro* and affect its structural stability, supporting their detrimental role on HBsAg secretion. In this setting, genotypic testing can help in optimizing the clinical interpretation of HBsAg levels in HBV genotype D and in providing information on viral pathogenicity and disease progression.

## Supplementary Material

Supplemental Material

## References

[1] WHO. Global Hepatitis Report, 2017. (2017). http://apps.who.int/iris/bitstream/handle/10665/255016/9789241565455-eng.pdf;jsessionid=61EC78993CF68B73EEADAA884E757FF0?sequence=1 (accessed 30 Aug 2018).

[2] Lampertico P, Agarwal K, Berg T, et al. EASL 2017 Clinical Practice Guidelines on the management of hepatitis B virus infection. J Hepatol. 2017;67:370–398. doi: 10.1016/j.jhep.2017.03.021 28427875

[3] Brunetto MR, Oliveri F, Colombatto P, et al. Hepatitis B surface antigen serum levels help to distinguish active from inactive hepatitis B virus genotype D carriers. Gastroenterology. 2010;139:483–490. doi: 10.1053/j.gastro.2010.04.052 20451520

[4] Riveiro-Barciela M, Bes M, Rodríguez-Frías F, et al. Serum hepatitis B core-related antigen is more accurate than hepatitis B surface antigen to identify inactive carriers, regardless of hepatitis B virus genotype. Clin Microbiol Infect. 2017;23:860–867. doi: 10.1016/j.cmi.2017.03.003 28288829

[5] Lin CL, Kao JH. Hepatitis B virus genotypes and variants. Cold Spring Harb Perspect Med 2015. doi:10.1101/cshperspect.a021436. PMC4448583 25934462

[6] Sozzi V, Walsh R, Littlejohn M, et al. *In vitro* studies show that sequence variability contributes to marked variation in hepatitis B virus replication, protein expression, and function observed across genotypes. J Virol. 2016;90:10054–10064. doi: 10.1128/JVI.01293-16 27512071 PMC5105644

[7] Rajoriya N, Combet C, Zoulim F, et al. How viral genetic variants and genotypes influence disease and treatment outcome of chronic hepatitis B. Time for an individualised approach? J Hepatol. 2017;67:1281–1297. doi: 10.1016/j.jhep.2017.07.011 28736138

[8] Bruss V. Hepatitis B virus morphogenesis. World J Gastroenterol. 2007;13:65. doi: 10.3748/wjg.v13.i1.65 17206755 PMC4065877

[9] Blumberg B. Australia antigen and the biology of hepatitis B. Science (80-). 1977;197:17–25. doi: 10.1126/science.325649 325649

[10] Hu J, Liu K. Complete and incomplete hepatitis B virus particles: formation, function, and application. Viruses. 2017. doi:10.3390/v9030056. PMC5371811 28335554

[11] Peiffer KH. Divergent preS sequences in virion-associated Hepatitis B virus genomes and subviral HBV surface antigen particles from HBV e antigen-negative patients. J Infect. 2018;218(1):114–123. 10.1093/infdis/jiy119 29528436

[12] Bruss V, Ganem D. Mutational analysis of hepatitis B surface antigen particle assembly and secretion. J Virol. 1991;65:3813–3820. doi: 10.1128/JVI.65.7.3813-3820.1991 2041095 PMC241412

[13] Stirk HJ, Thornton JM, Howard CR. A topological model for hepatitis B surface antigen. Intervirology. 1992. doi:10.1159/000150244. 1500275

[14] Khan N, Guarnieri M, Ahn SH, et al. Modulation of Hepatitis B virus secretion by naturally occurring mutations in the S gene. J Virol. 2004;78:3262–3270. doi: 10.1128/JVI.78.7.3262-3270.2004 15016847 PMC371066

[15] Wang H-C, Huang W, Lai M-D, et al. Hepatitis B virus pre-S mutants, endoplasmic reticulum stress and hepatocarcinogenesis. Cancer Sci. 2006;97:683–688. doi: 10.1111/j.1349-7006.2006.00235.x 16863502 PMC11158693

[16] Salpini R, Colagrossi L, Bellocchi MC, et al. Hepatitis B surface antigen genetic elements critical for immune escape correlate with hepatitis B virus reactivation upon immunosuppression. Hepatology. 2015;61:823–833. doi: 10.1002/hep.27604 25418031

[17] Svicher V, Gori C, Trignetti M, et al. The profile of mutational clusters associated with lamivudine resistance can be constrained by HBV genotypes. J Hepatol. 2009. doi:10.1016/j.jhep.2008.07.038. 19041149

[18] van Hemert FJ, Zaaijer HL, Berkhout B, et al. Mosaic amino acid conservation in 3D-structures of surface protein and polymerase of hepatitis B virus. Virology. 2008;370:362–372. doi: 10.1016/j.virol.2007.08.036 17935747

[19] Yang J, Yan R, Roy A, et al. The I-TASSER suite: protein structure and function prediction. Nat Methods. 2015;12:7–8. doi: 10.1038/nmeth.3213 PMC4428668 25549265

[20] Roy A, Kucukural A, Zhang Y. I-TASSER: a unified platform for automated protein structure and function prediction. Nat Protoc. 2010;5:725–738. doi: 10.1038/nprot.2010.5 20360767 PMC2849174

[21] Yang J, Zhang Y. I-TASSER server: new development for protein structure and function predictions. Nucleic Acids Res. 2015;43:W174–W181. doi: 10.1093/nar/gkv342 25883148 PMC4489253

[22] Quan L, Lv Q, Zhang Y. STRUM: structure-based prediction of protein stability changes upon single-point mutation. Bioinformatics. 2016;32:2936–2946. doi: 10.1093/bioinformatics/btw361 27318206 PMC5039926

[23] Zhang F, Tang X, Garcia T, et al. Characterization of contrasting features between hepatitis B virus genotype A and genotype D in small envelope protein expression and surface antigen secretion. Virology. 2017;503:52–61. doi: 10.1016/j.virol.2017.01.009 28126637 PMC5325793

[24] Kuhnhenn L, Jiang B, Kubesch A, et al. Impact of HBV genotype and mutations on HBV DNA and qHBsAg levels in patients with HBeAg-negative chronic HBV infection. Aliment Pharmacol Ther. 2018;47:1523–1535. doi: 10.1111/apt.14636 29637585

[25] Freitas N, Cunha C, Menne S, et al. Envelope proteins derived from naturally integrated Hepatitis B virus DNA support assembly and release of infectious hepatitis delta virus particles. J Virol. 2014. doi:10.1128/jvi.00430-14. PMC4019103 24623409

[26] Liaw YF. Clinical utility of HBV surface antigen quantification in HBV e antigen-negative chronic HBV infection. Nat. Rev. Gastroenterol. Hepatol. 2019;16:631–641. doi: 10.1038/s41575-019-0197-8 31477873

[27] Ogata M, Hino S-I, Atsushi S, et al. Autophagy is activated for cell survival after endoplasmic reticulum stress. Mol Cell Biol. 2006;26:9220–9231. doi: 10.1128/MCB.01453-06 17030611 PMC1698520

[28] Sir D, Tian Y, Chen W, et al. The early autophagic pathway is activated by hepatitis B virus and required for viral DNA replication. PNAS. 2010;107:4383–4388. doi: 10.1073/pnas.0911373107 20142477 PMC2840127

[29] Xie M, Yang Z, Liu Y, et al. The role of HBV-induced autophagy in HBV replication and HBV related-HCC. Life Sci. 2018;205:107–112. doi: 10.1016/j.lfs.2018.04.051 29709654

[30] Wu C-C, Chen Y-S, Cao L, et al. Hepatitis B virus infection: defective surface antigen expression and pathogenesis. World J Gastroenterol. 2016;24:3488–3499. doi: 10.3748/wjg.v24.i31.3488 PMC6102499 30131655

[31] Wooddell CI, Yuen MF, Chan HLY, et al. Rnai-based treatment of chronically infected patients and chimpanzees reveals that integrated hepatitis b virus DNA is a source of hbsag. Sci Transl Med. 2017;9; doi:10.1126/scitranslmed.aan0241. PMC5830187 28954926

[32] Revill P, Testoni B, Locarnini S, et al. Global strategies are required to cure and eliminate HBV infection. Nat. Rev. Gastroenterol. Hepatol. 2016;13:1–10. doi: 10.1038/nrgastro.2016.7 26907881

[33] Cornberg M, Wong VW-SS, Locarnini S, et al. The role of quantitative hepatitis B surface antigen revisited. J Hepatol. 2017;66:398–411. doi: 10.1016/j.jhep.2016.08.009 27575311

