# Supplementary material for: Key mutations in the C-terminus of the HBV surface glycoprotein correlate with lower HBsAg levels in vivo, hinder HBsAg secretion in vitro and reduce HBsAg structural stability in the setting of HBeAg-negative chronic HBV genotype-D infection
Source: Emerg Microbes Infect. 2020 May 13;9(1):928–39. doi: 10.1080/22221751.2020.1757998 (PMC7269061; doi:10.1080/22221751.2020.1757998)
Supplement: Supplemental Material [file TEMI_A_1757998_SM0422.docx]

**Supplementary material**

***Protocol for HBsAg sequencing***

HBV-DNA was extracted using a commercially available kit (QIAmp DNA blood mini-kit, Qiagen Inc., USA), and then amplified with Amplitaq-Gold polymerase using the following primer pairs: F1-5’GGTCACCATATTCTTGGGAA and R1-5’GTGGGGGTTGCGTCAGCAAA. PCR conditions were: one cycle at 93 °C for 12 min, 40 cycles (94 °C 50 s, 57 °C 50 s, 72 °C 1 min and 30 s), and a final cycle at 72 °C for 10 min. For samples with serum low HBV-DNA, 2 additional eminested-PCR were performed, starting from the same first amplicon: eminested_1 used the following primer pairs (F1-5’GGTCACCATATTCTTGGGAA and R2-GAGGACAAACGGGCAACATACCTT and eminested-2 used F2-GTTGACAAGAATCCTCACAATA and R1-5’GTGGGGGTTGCGTCAGCAAA. Both hemi-nested-PCRs conditions were: one cycle at 93 °C for 12 min, 35 cycles (94 °C 50 s, 56 °C 50 s, 72 °C 1 min), and a final cycle at 72 °C for 10 min. PCR-products were purified and sequenced by using eight different overlapping sequence-specific primers, a BigDye terminator v. 3.1 cycle sequencing kit (Applied-Biosystems FosterCity, CA) and an automated sequencer (Genetic Analyzer 3130XL). The sequences were analyzed using SeqScape-v.2.5 software. The quality endpoint for each individual gene was ensured by a coverage of the S gene sequence by at least two segments. Sequences having a mixture of wild-type and mutant residues at single positions were considered to have the mutant(s) at that position.

***Phylogenetic analysis***

HBV genotypes were determined by constructing phylogenetic tree using the Neighbor-Joining (NJ) method (Saitou and Nei, 1987). Distances were calculated using MEGA 6 based on the Kimura-2 parameter (K2P) model (Kimura, 1980). The reliability of the branching orders was assessed by bootstrap analysis of 1000 replicates.

**Covariation analysis among mutations**

The binomial-correlation coefficient (phi) was calculated for each pair of mutations to assess the strength of co-variation among mutations. Fisher’s exact test was performed to assess statistically significant pairs of mutations. Covariation analysis was also repeated in the subset of 91 patients with HBeAg-negative genotype D infection.

In order to support the correlation of the identified pairs of HBsAg C-terminus mutations with HBsAg<1,000IU/ml, a logistic regression analysis was performed in the set of 228 patients considering the following variables: gender, age, HBV-DNA, ALT, presence of >1 pair of HBsAg C-terminus mutations associated with HBsAg<1,000IU/ml. After stepwise elimination for optimized Akaike information criterion (AIC), only variables showing a p-value <0.05 in univariate analysis were included in the multivariate analysis.

***Plasmid used in vitro experiments***

The plasmid used in our experiments was the pEXPR-IBA44 vector, in which the gene encoding the small-sized HBV genotype D surface glycoprotein (HBsAg, aa 1-226, derived from the pCHsAg vector) was cloned by using the restriction enzymes XhoI and HindIII (IBA, Göttingen, Germany).

In this plasmid, HBsAg expression is under the control of the human cytomegalovirus (CMV) immediate-early promoter. In this plasmid, the strep-tag is upstream the gene encoding HBsAg, thus resulting at the N-terminus of the corresponding protein.

The Streptavidin-tag version II, used in our experiments, is a short (eight-residues, Trp-Ser-His-Pro-Gln-Phe-Glu-Lys) peptide sequence, biologically inert, proteolytically stable, that has been widely used in several papers for protein detection and purification (Schmidt and Skerra, 2007; Skerra and Schmidt, 2000). These previous studies have largely demonstrated that it does not affect protein secretion and does not interfere with protein folding (Schmidt and Skerra, 2007). For this reason, the Strep-tag is especially and widely suited to isolate proteins and to study their structures and functions.

***Cell culture and transfections***

HepG2 cells were grown in a 37°C humidified atmosphere containing 5% CO2, using Dulbecco’s modified Eagle’s medium (DMEM) (Life Technologies, Inc., Gaithersburg, MD) supplemented with 10% fetal bovine heat-inactivated serum and with 100 U/ml penicillin + 100µg/ml streptomycin + 2 mM L-glutamine. HepG2 cells were transiently transfected with the pEXPR-Strep- HBsAg containing either *wt* and HBsAg mutants by using TransIT-X2 Transfection Reagent (Mirus Bio LLC, USA). After 72 hours post transfection, cell supernatants were collected, clarified by centrifugation at 4000g for 5min, applied on StrepMAB-Immo coated microplates (IBA) and quantified as previuosly described ^10^. For each sample, at least 3 independent transfection experiments, each led in duplicate, were performed. Transfection efficiency was monitored by co-transfection of a vector expressing GFP, which can be measured by cytofluorimetric assay. Results were expressed as percentage of production compared to *wt* (considered as 100%).

***In silico prediction of HBsAg structure***

The impact of aa substitutions on HBsAg stability was investigated by using STRUM, a well-consolidated and accurate method, that allows to evaluate changes in ∆∆G in presence of specific mutations. Indeed, differently from previous approaches (only based on sequence analyses), STRUM has been trained on a wide set of experimentally characterized mutatiosn (N=3,421) obtained from 150 proteins, thus resulting in an important improvement in the accuracy of ∆∆G prediction (Quan et al., 2016).

**References**

Kimura, M., 1980. A simple method for estimating evolutionary rates of base substitutions through comparative studies of nucleotide sequences. J. Mol. Evol. 16, 111–120. https://doi.org/10.1007/BF01731581

Quan, L., Lv, Q., Zhang, Y., 2016. STRUM: structure-based prediction of protein stability changes upon single-point mutation. Bioinformatics 32, 2936–46. https://doi.org/10.1093/bioinformatics/btw361

Saitou, N., Nei, M., 1987. The neighbor-joining method: a new method for reconstructing phylogenetic trees. Mol. Biol. Evol. 4, 406–25. https://doi.org/10.1093/oxfordjournals.molbev.a040454

Schmidt, T.G., Skerra, A., 2007. The Strep-tag system for one-step purification and high-affinity detection or capturing of proteins. Nat. Protoc. 2, 1528–1535. https://doi.org/10.1038/nprot.2007.209

Skerra, A., Schmidt, T.G., 2000. Use of the Strep-Tag and streptavidin for detection and purification of recombinant proteins. Methods Enzymol. 326, 271–304. https://doi.org/10.1016/s0076-6879(00)26060-6

**Table S1. Multivariate linear regression model to define factors associated with HBsAg (expressed as log_10_IU/ml)**

| **Variables^a^** | **Estimate** | **Std. Error** | **P value** |
| --- | --- | --- | --- |
| Interecept | 3.73 | 0.25 | <0.0001 |
| Genotype A vs. D^b^ | 0.52 | 0.06 | <0.0001 |
| Genotype E vs. D^b^ | 0.35 | 0.15 | 0.020 |
| Female vs Male | -0.10 | 0.09 | 0.22 |
| Age (per 1 year increase) | -0.02 | 0.0031 | <0.0001 |
| Serum HBV-DNA (per 1logIU/ml increase) | 0.16 | 0.050 | 0.0016 |
| ALT (per 1 U/L increase) | 0.000099 | 0.00031 | 0.75 |
| Status of HBV infection^c^ | -0.17 | 0.12 | 0.15 |

^a^Univariate and multivariate linear regression model was led in 323 HBeAg-negative patients infected with HBV genotype D (N=228), A (N=65), E (N=30).

^b^The estimates indicate that the mean difference between genotype A versus genotype D is +0.50logIU/ml, while the mean difference between genotype E versus genotype D is +0.33logIU/ml, after correcting for patients’ demographics, serum HBV-DNA and ALT.

^c^ Having a status of HBeAg-negative infection (defined as serum HBV-DNA<2,000IU/ml and persistently normal transaminases) versus not having HBeAg-negative infection

**Table S2. Association between HBsAg C-terminus mutations correlated with HBsAg <1000 IU/mL and other mutations in HBsAg C-terminus in the subset of patients with HBeAg-negative patients (N=91)**

| **Mutations associated**  **with HBsAg<1000 IU/ml** | **Correlated**  **mutations** | **Phi^a^** | **P-Value^a^** | **N (%) of the pairs of mutations in HBsAg** | |
| --- | --- | --- | --- | --- | --- |
|  |  |  |  | **<1,000IU/ml^b^** | **>1,000IU/ml^b^** |
| **V190A** | **F220L** | 0.43 | 0.001 | 5 (9.6) | 0 |
| **Y206F** | **V194A** | 0.20 | 0.09 | 3 (5.8) | 0 |
|  | **M197T** | 0.40 | 0.007 | 3 (5.8) | 0 |
|  | **S204T** | 0.20 | 0.128 | 2 (3.8) | 0 |
|  | **S210R** | 0.49 | <0.001 | 6 (11.5) | 0 |
| **S204N** | **L205P** | 0.38 | 0.001 | 4 (7.7) | 1 (2.6) |
| **S210N** | **F220L** | 0.41 | 0.002 | 4 (7.7) | 0 |

^a^ Binomial correlation coefficient (Phi) was calculated to assess the strength of association for each pair of mutations identified. Statistically significant differences were assessed by Fisher Exact Test.

^b^ The prevalence of the pairs of mutations was calculated in the group of 52 patients with HBsAg <1,000IU/ml and 39 patients with HBsAg >1,000IU/ml.

| **Pairs of HBsAg mutations associated with HBsAg levels< 1,000 IU/ml** | **N (%) of the pairs of HBsAg mutations^a^** | **Correspondent**  **mutations in RT^b^** | **N (%) of the pairs of mutations in RT^c^** |
| --- | --- | --- | --- |
|  |  |  |  |
| **sV190A + sF220L** | 5 (6.5) | **rtNone + rtL229M** | 5 (100) |
| **sY206F + sV194A** | 5 (6.5) | **rtQ215H + rtNone** | 3 (60) |
|  |  | **rtV214A + rtNone** | 2 (40) |
| **sY206F + sM197T** | 4 (5.2) | **rtV214A + rtNone** | 2 (50) |
|  |  | **rtQ215H + rtNone** | 2 (50) |
| **sY206F + sS204T** | 4 (5.2) | **rtV214A + rtK212N** | 2 (50) |
|  |  | **rtQ215H + rtH212N** | 2 (50) |
| **sY206F + sS210R** | 8 (10.4) | **rtQ215H + srtS219A** | 4 (50) |
|  |  | **rtV214A + S219A** | 2 (25) |
|  |  | **rtQ215S + S219A** | 1 (12.5) |
|  |  | **rtNone + S219T** | 1 (12.5) |
| **sS204N + sL205P** | 5 (6.5) | **rtNone + rtNone** | 5 (100) |
| **sS210N + sF220L** | 4 (5.2) | **rtNone + rtL229M** | 2 (50) |
|  |  | **rtE218D + rtL229M** | 1 (25) |
|  |  | **rtS219A + rtL229M** | 1 (25) |

**Table S3. Correspondence between the pairs of HBs C-terminus mutations correlated with HBsAg <1000 IU/ml and aa substitutions in RT region**

**^a^** The prevalence of the pairs of HBsAg mutations associated with HBsAg<1,000 IU/ml was calculated in the 228 HBeAg negative genotype D-infected patients

**^b^** The correspondence between the pairs of HBsAg mutations associated with HBsAg<1,000 IU/ml and the RT mutations has been reported in the same order

**^c^** The prevalence of the pairs of RT mutations was calculated on the overall patients carrying each corresponding pair of HBsAg mutation associated with HBsAg<1,000 IU/ml

**Table S4. Prevalence of the identified mutations in genotype A and E**

| **Mutations associated with HBsAg<1,000 IU/ml** | **N (%) genotype A, N=65** | **N (%) genotype E, N=30** |
| --- | --- | --- |
| V190A | 0 (0) | 0 (0.0) |
| V190A+F220L | 0 (0) | 0 (0.0) |
| S204N | 12 (18.5) | 6 (20.0) |
| S204N+L205P | 0 (0) | 0 (0.0) |
| Y206F | 1 (1.5) | 1 (3.3) |
| Y206F+V194A | 1 (1.5) | 0 (0.0) |
| Y206F+M197T | 0 (0) | 0 (0.0) |
| Y206F+204T | 0 (0) | 0 (0.0) |
| Y206F+S210R | 0 (0) | 0 (0.0) |
| S210N | 0 (0) | 1 (3.3) |
| S210N+F220L | 0 (0) | 0 (0.0) |

**Figure S1**

**
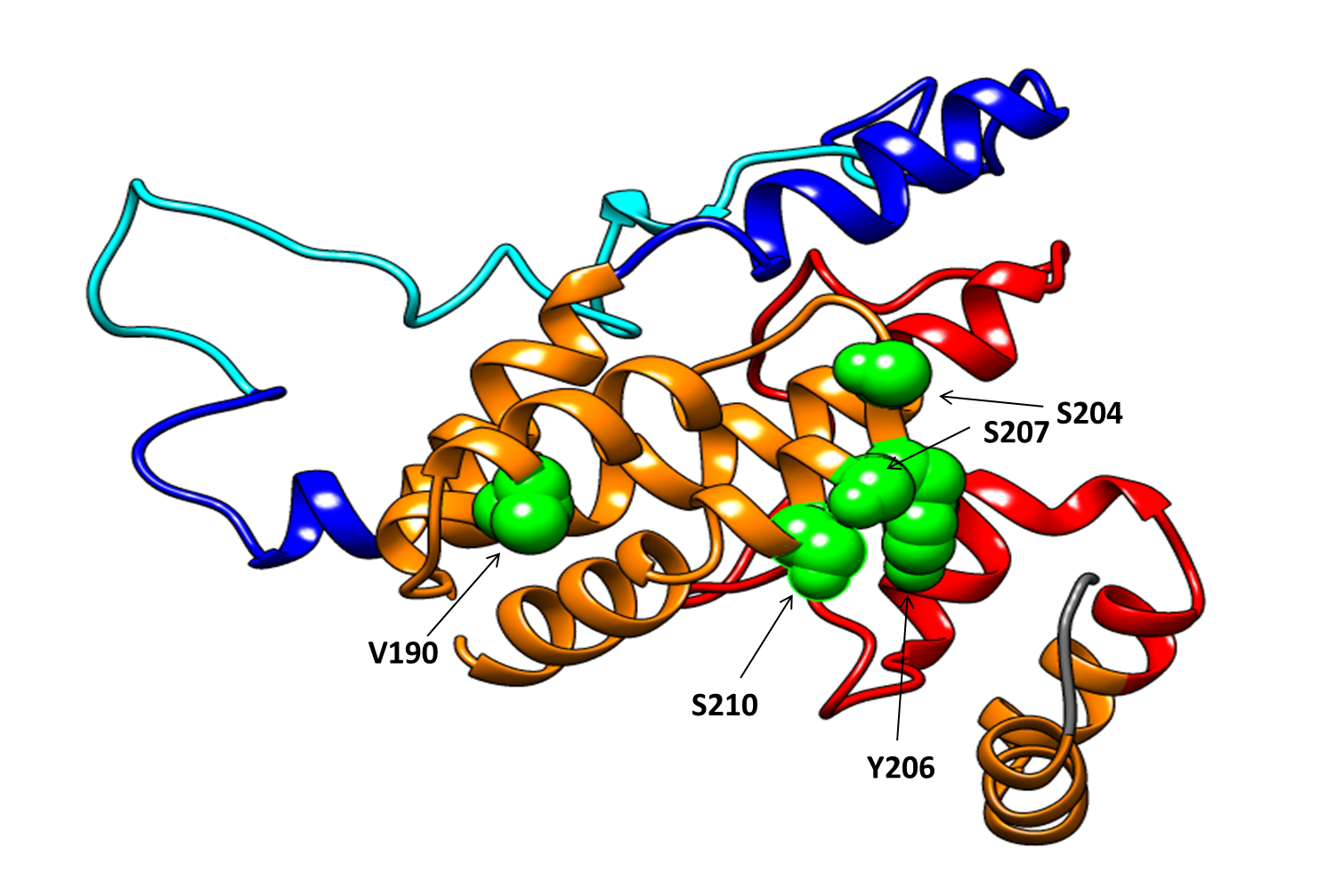
**

**Figure S1.** Localization of amino acid residues associated with low HBsAg levels (< 1,000 IU/ml). The 3D model of HBsAg was generated by I_TASSER. Functional HBsAg domains are color-coded in the “D-consensus” protein: amino acids not assigned (1-7, gray), transmembrane signals (8-22, 80-98, orange) and membrane-embedded C-terminus (169-226, orange), cytosolic loop (23-79, red), major hydrophilic region (99-169, dark-blue), the a-determinant region (110-140, cyan). The wild-type amino acid is reported for each position: Valine, V; Serine, S; Tyrosine, Y.

**Figure S2**

**
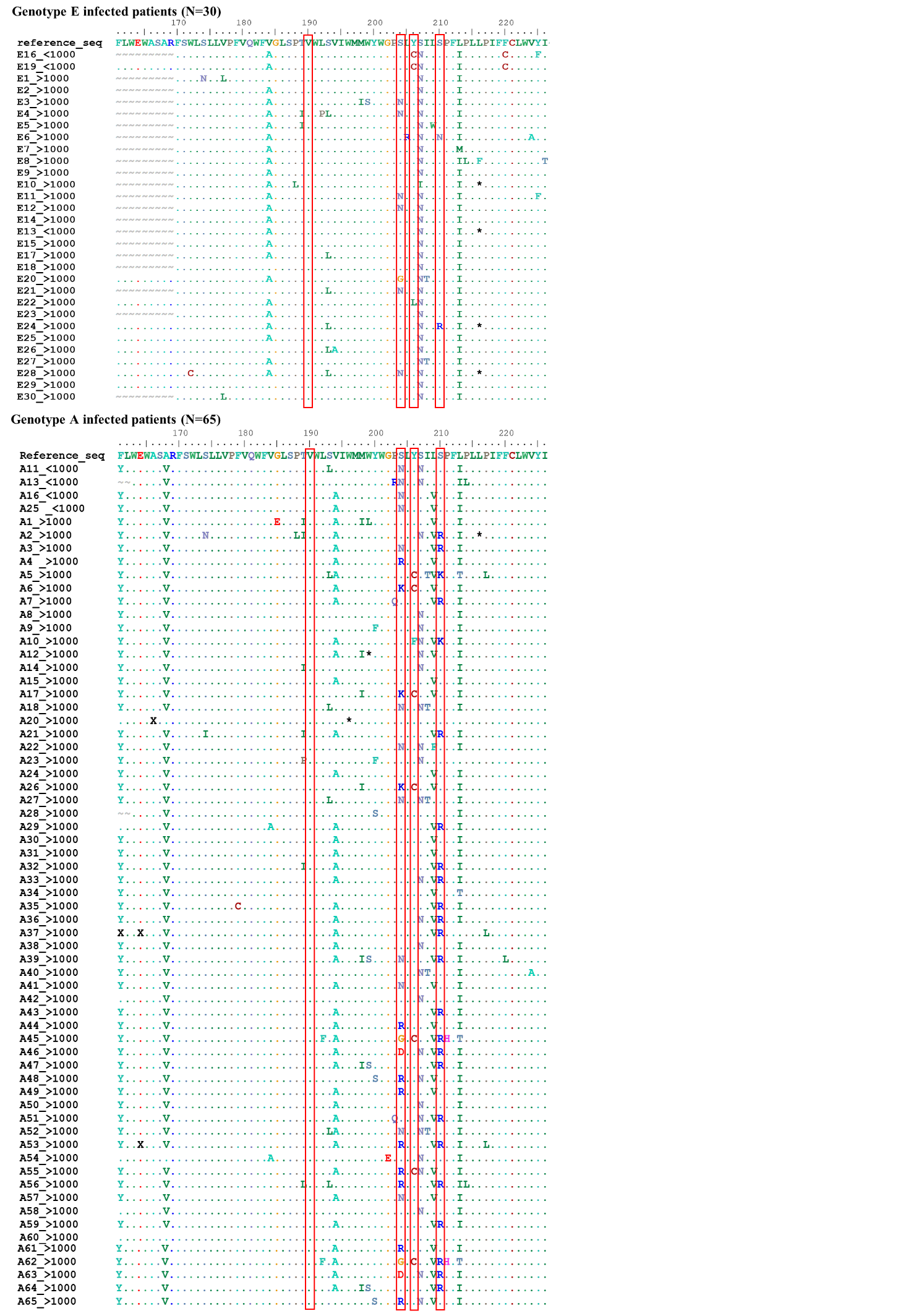
**

**Figure S2.** Raw sequencing data (in Bioedit format) for both genotype A and E, highlighting the mutations in the C-terminal HBsAg domain in patients with HBsAg<1000 IU/ml and >1000 IU/ml.
